# Magnitude and determinants of delivery complications among women in selected hospitals of West Shoa, Oromia, Ethiopia: A multicentered cross-sectional study

**DOI:** 10.1371/journal.pone.0346778

**Published:** 2026-04-22

**Authors:** Teka Girma, Desalegn Wirtu, Gudina Egata

**Affiliations:** 1 School of Public Health, Institute of Health, Wallaga University, Nekemte City, Ethiopia; 2 Department of Public Health, College of Health Sciences, and Referral Hospital, Ambo University, Ambo City, Ethiopia; 3 Department of Nutrition and Dietetics, School of Public Health, College of Health Sciences, Addis Ababa University, Addis Ababa City, Ethiopia; Debre Tabor University, ETHIOPIA

## Abstract

**Background:**

The occurrence of Complications during delivery is a significant contributor to the health of the mothers and neonates, particularly in low-resource settings like Ethiopia. Assessing the magnitude and factors associated with it is essential for improving obstetric outcomes and having a healthy family. This study aims to identify the magnitude of delivery complications and associated factors among pregnant women who gave birth at West Shoa Hospitals, Oromia, Ethiopia.

**Methods:**

A facility-based cross-sectional study design was conducted among 573 pregnant women who gave birth in the randomly selected hospitals, in West Shoa, from 1/7/2023–30/3/ 2024. Data were collected through structured face-to-face interviews and from the extraction of medical records. Binary logistic regression analysis was performed to identify factors associated with delivery complications, and variables with a p- p-value <0.25 in the bivariate analysis were included in the multivariable logistic regression model, and the statistical significance was determined at a p-value <0.05.

**Results:**

The proportion of delivery complications was 30%(95%CI:26.4–33.9). In the multivariable logistic regression analysis, depression increases with 1.5 times the odds of complication (AOR:1.5; 95% CI (1.06–1.6); P = 0.012). Maternal hemoglobin level was significantly associated with higher odds of delivery complications. Women with low haemoglobin had 6% lower odds of experiencing delivery complications compared with non-anaemic women (AOR:0.94; 95% CI (0.93–0.97); P = 0.01). Women with foetal malpresentation had significantly increased odds of experiencing delivery complications compared with cephalic presentation (AOR = 6.97, 95% CI:1.79–26.68); P = 0.001.HIV positive women exhibited a significantly lower risk of delivery complications compared to HIV negative women (AOR = 0.37;95% CI:0.19–0.72; P = 0.004).

**Conclusion:**

Complications during deliveries are high in the study area, with depression during pregnancy, having, and foetal malpresentation identified as predictor variables. Interventions should focus on early screening and management of maternal anaemia and mental health, as well as enhanced antenatal surveillance for foetal presentation.

## Introduction

Maternal mortality remains a critical global health priority, particularly in low and middle-income countries (LMICs) where maternal mortality rates are unacceptably high. The World Health Organization (WHO) reported that around 260,000 women died from pregnancy-related issues in 2023, with about 70% of these deaths from sub-Saharan Africa. Despite about forty percent in maternal deaths between 2000 and 2023, progress has lagged since 2016, and current aid cuts threaten to reverse these gains [1].

Pregnancy is a transformative period that requires optimal maternal health to ensure positive outcomes for both the mother and the newborn. Body mass index (BMI) before pregnancy gives a baseline assessment of maternal nutritional status, while gestational weight gain (GWG) reflects physiological and dietary changes necessary for foetal development and maternal adaptation during [2]. Deviation from the recommended BMI and GWG guidelines predisposes women to serious complications during delivery, including pregnancy-induced hypertension, gestational diabetes mellitus, prolonged Labour, and CS delivery [3].

The Sustainable Development Goal(SDG) 3.1 aims to reduce the global maternal mortality ratio to below 70% per 100,000 live births by the year 2030 [4]. Globally, efforts to reduce maternal mortality have primarily focused on addressing the direct medical causes of death, particularly during pregnancy and childbirth [5]. However, the WHO advises that all women should receive high-quality, evidence-based care during childbirth without considering health care settings. To effectively implement these recommendations, the national and regional health policies and clinical protocols must be aligned and strengthened [6]. In Ethiopia, the Federal Ministry of Health introduced the national Maternal Death Surveillance and Report (MDSR) system in to improve the quality of maternal health care, with particular emphasis on the pregnancy, Labour, and postpartum periods [7]. Globally, there is a dual malnutrition, both undernutrition and overnutrition, and from this problem, women of reproductive age are significantly challenged. According to the World Health Organization [8], obesity rates among women have doubled in the past three decades, while undernutrition remains prevalent in low-income countries. Similarly, inappropriate GWG, whether excessive or inadequate, is associated with adverse maternal and neonatal outcomes, underscoring the importance of weight management during pregnancy [9,10].

Maternal mortality is a significant health problem in Africa, with serious consequences [11]. Preventable maternal death on the continent continues to be a significant issue, with a major contribution from complications like bleeding, high blood pressure, sepsis, and prolonged labour [12]. Eclampsia and haemorrhage are the primary factors leading to maternal mortality in Africa [1]. Premature rupture of membranes also complicates 5–10% of all deliveries [13]. The magnitude of premature rupture of membranes among preterm births was 30% worldwide, and can result in complications like birth asphyxia and respiratory distress in babies due to a sudden decrease in amniotic fluid levels [14]. Obstetric haemorrhage is the main cause of death, usually associated with a shortage of resources [15].

Pre-pregnancy body mass index is categorized into four categories according to the WHO: Underweight (<18.5 kg/m²), normal weight (18.5–24.9 kg/m²), Overweight (25–29.9 kg/m²), and Obese (≥30 kg/m²). Literature shows that each category is associated with different health risks: Underweight women are at risk of preterm, IUGR, and low birth weight [9]. Whereas, overweight pregnant women and obese women face risks of gestational high blood pressure, eclampsia, and caesarean section delivery, as well as the risk of delivery of macrosomia [10].

Nowadays, there is existing literature that shows that complications during delivery are highly related to gestational weight, which has not received emphasis. The dual burden of malnutrition, characterized by the coexistence of underweight and obesity, has brought disparities in maternal outcomes. Studies indicate that maternal overweight increases the risk of delivery complications like cs delivery and prolonged labour [10]. On the contrary, underweight women are prone to experience complications like having a preterm baby and foetal growth restriction [9].

High gestational weight gain is linked to hypertensive disorders, pregnancy-induced DM, and macrosomia [2]. Insufficient gestational weight gain, on the other hand, is associated with preterm delivery and low birth [16] where both cases increase the chance of delivery complications, placing additional burden on the healthcare system and consuming more resources [8].

In Ethiopia, even though a lot has been made to reduce maternal and neonatal mortality to meet the global commitments, delivery complications remain at a persistently high rate and pose a public health challenge, particularly in rural and semi-urban settings like West Shoa Zone. Most existing studies in Ethiopia and other similar countries focus on maternal health broadly; only a few studies have explored the predictors of intrapartum complications using both clinical and psychosocial issues, such as mental health status, nutritional indicators, in combination. Furthermore, data on delivery outcome linked to psychosocial and obstetric history at the zonal regional level are limited.

This study addresses these gaps by providing context-specific, evidence-based insights into the magnitude and determinants of delivery complications in the West Shoa zone. This study addresses these gaps by providing context-specific, evidence-based insights into the magnitude and determinants of delivery complications in the West Shoa Zone. It also contributes to literature by highlighting often overlooked factors like antenatal depression and other obstetric-related variables. The findings of the study can also guide the local health policy-makers, maternal health programmers, and clinicians in designing targeted, specific interventions such as mental health screening, improving anaemia management and prevention, monitoring weight gain, and foetal presentation. Ultimately, the study will support national and global efforts to improve maternal outcomes and progress towards Sustainable Development Goal (SDG) targets related to maternal health.

### Methods and materials

#### Study setting and period.

The investigation was conducted in government hospitals found in the study area for nine months from 1/7/ 2023, to 30/3/, 2024 [17].

### Study design and populations

A multicentre cross-sectional study was employed to assess the proportion of delivery complications and their associated factors among postpartum women. The study was anchored in a positivist research paradigm, which assumes that phenomena are measurable, observable, and governed by systematic data collection and quantitative analysis to identify associations between independent variables and delivery complications.

The source population consisted of all postpartum women who delivered in public hospitals of the West Shoa during the study period. The study population includes postpartum women who delivered in the randomly selected hospitals, had attended antenatal care (ANC) in those facilities, and met the eligibility criteria. Data were obtained through a combination of structured interviews and the extraction of clinical information from medical and Laboratory records to ensure accuracy and completeness.

## Eligibility criteria

### Inclusion criteria

- Postpartum women who received ANC follow-up in the respective study facilities
- Permanent residents of the study area during the pregnancy period
- Women with complete clinical, medical, and Laboratory documentation in their obstetric records

### Exclusion criteria

- Women delivered outside of the study facilities and subsequently admitted during the data collection period

The main outcome variable was the presence of delivery complications, while additional socio-demographic variables, reproductive history, physical activity, dietary diversity, depression during pregnancy, and clinical variables were extracted from the medical records for analytical purposes.

### Sample size and sampling procedure

The sample size was calculated using a single proportion formula, based on a 95% confidence level, prevalence of delivery complications of 33.86% as reported from the previous study [18] and a 5% margin of error, using the formula:


$$n=\frac{z^{\text{2}}\;p(\text{1}-p)}{e^{\text{2}}}$$

Considering clustering across facilities design effect was 1.5, and a 10% non-response rate, which yielded 573 a final sample size.

**Fig 1:**
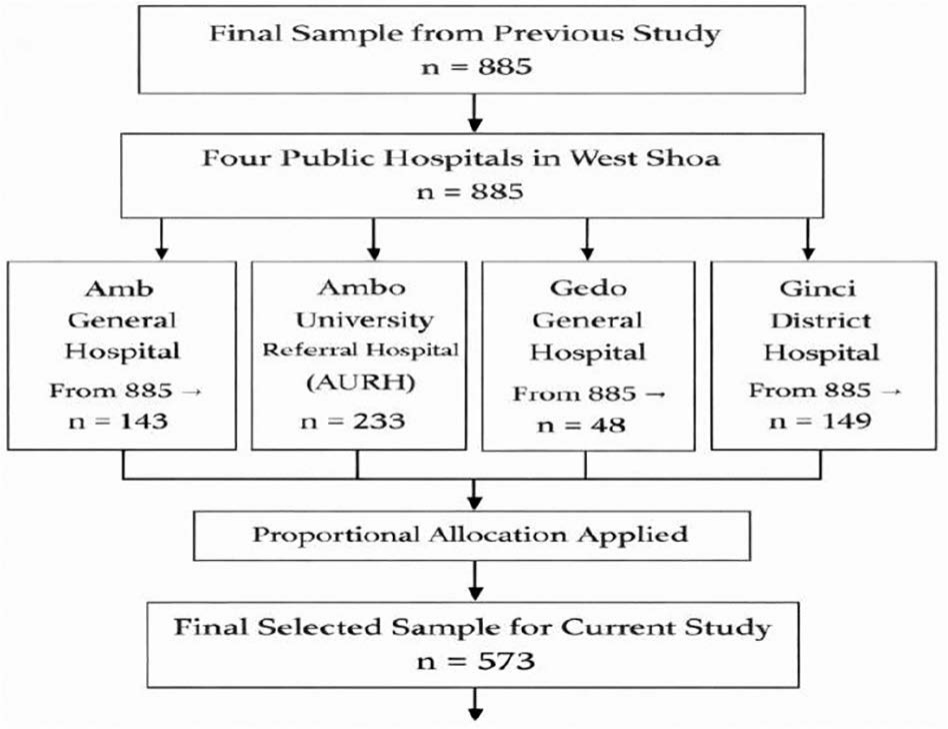
Schematic representation of the sampling procedure for this study.

A multi-stage sampling strategy was applied in the first stage; four hospitals were selected from the nine hospitals found in the zone through simple random sampling. In the second stage, the sample size was allocated to each hospital in proportion to the monthly number of deliveries, ensuring representativeness. Within each hospital, systematic random sampling was used to select eligible postpartum women(Fig 1). The sampling interval (K) for each facility was calculated by dividing the monthly deliveries by the allocated sample size. Data collectors, supported by facility staff, enrolled participants who met the inclusion criteria at predetermined intervals

### Operational definitions and measurements

• Delivery complications: defined as any adverse maternal, foetal, or neonatal conditions occurring during Labour or childbirth as diagnosed by skilled birth attendants or documented in medical records (preeclampsia, prolonged Labour, postpartum haemorrhage (PPH), retained placenta, malpresentation, premature rupture of membrane, and antepartum haemorrhage (APH).

## Study variables

### Dependent variable

#### Delivery complications (Yes/no).

The study’s dependent variable was the presence of obstetric complications during delivery. This variable was categorized dichotomously: women who experienced at least one complication—such as severe bleeding, rupture of membranes accompanied by no labour contractions for more than 24 hours, premature rupture of membranes before nine months of gestation, malposition or malpresentation, prolonged labour exceeding 12 hours, or convulsions/fits, were coded as 1. Those who did not experience any of these complications were coded as 0. The occurrence of complications was based on women’s self-reports and was verified through medical record review or documented clinical diagnosis.

### Independent Variables

Sociodemographic characteristics: age, height, weight, residence, religion, educational attainment, marital status, occupation, monthly income, and cohabitation status.

Gestational weight gain: -categorized as insufficient, adequate, or excessive.

Birth outcome (preterm birth, still birth, healthy baby, NICU admission)

Birth weight (low birth weight, normal weight, or macrosomia)

Mode of delivery (spontaneous vaginal delivery, caesarean section, or instrumental delivery)

### Data collection procedures

An inclusive, organised, structured data collection instrument was developed following an extensive review of evidence on maternal and neonatal health outcomes. The initial questionnaire was drafted in English, reviewed by language experts, and subsequently translated into Afan Oromo, the regional official working language. To ensure linguistic fidelity and conceptual stability, a blinded back translation into English was undertaken, and discrepancies were resolved by consensus among bilingual experts. The final instrument encompassed multiple domains, including sociodemographic characteristics, reproductive and obstetric history, dietary diversity, household food insecurity status, intimate partner violence exposure, and depression during pregnancy [17].

Main data were obtained through structured face-to-face interviews using an application platform, which minimizes error during data entry and timely validation checks, and facilitates consistent administration of survey items. Interviews were conducted by trained data collectors with extensive experience in maternal health data collection. The clinical and Laboratory data were abstracted from facility-based medical records using a standardized extraction sheet. Extracted variables included ultrasound-verified gestational age, serial blood pressure measurements, random blood glucose levels, haemoglobin concentration, and HIV serostatus. Maternal and neonatal outcomes documented by attending clinicians were also retrieved to corroborate self-reported information and eliminate potential bias.

### Data collection tools

A questionnaire was used to collect the data on socio-demographic characteristics, dietary diversity and food security, intimate partner violence, physical activity, and depression-related symptoms. Variables such as gestational age (ultrasound result), blood pressure, random blood sugar, anaemia status, and HIV status were obtained from the medical records of the women. The principal component analysis was employed to compute a wealth index from a set of household asset questions, such as electricity, refrigerator, table, chair, watch, phone, bed with mattress, electric bakery, car, house, improved water, and improved toilet, which was adapted from the Ethiopian demographic and health survey [19].

Mid-upper arm circumference (MUAC) was measured using an adult MUAC non-stretchable measuring tape, and the reading was taken to the nearest 0.1 cm. A MUAC measurement below 23 cm was categorized as low (or wasting), and above 23 cm was categorized as normal.

Perinatal depression symptoms were measured using the Edinburgh postnatal depression scale (EPDS) (114), which was already validated and contextualized in the local context, which is a ten-item questionnaire. It has been validated and used by many studies for detecting perinatal depression in Ethiopia [20].Household food insecurity was assessed using the Household Food Insecurity Access Scale (HFIAS). In each domain of the HFIAS, questions ask about anxiety and uncertainty; insufficient quality; and insufficient food intake and any physical consequences, with a recall period of four weeks (30 days) [21]Dietary diversity of the women was assessed using a minimum dietary diversity-women (MDD-W) set from the Food and Agriculture Organization (FAO) and USAID’s Food and Nutrition Technical Assistance III Project (FANTA) [22]. The food groups assessed in MDD-W include grains, white roots, tubers, and plantains; pulses; nuts and seeds; dairy; meat, poultry, and fish; eggs; vegetables; other vitamin A-rich fruits and vegetables; other vegetables; and other fruits. The MDD-W was a dichotomous indicator of whether or not women had consumed at least five out of ten defined food groups the previous day or night. The proportion of women who reach this minimum was used as a proxy indicator for higher micronutrient adequacy.

### Data quality assurance

To confirm methodological rigor, a multifaceted data quality assurance context was instituted. Data collectors with prior experience in survey implementation and clinical data extraction were recruited and underwent intensive training on the survey protocol, instrument content, standardized measurement protocols, electronic data entry procedures, and confidentiality safeguards. Supervisors took additional training on advanced data auditing and monitoring procedures. A pretest on 5% of the sample size was conducted in Guder Hospital to evaluate the clarity, cultural appropriateness, and psychometric reliability of the instrument. Revisions were incorporated based on pretest findings to optimize tool performance [17].

During the main data collection phase, daily supervisory checks were conducted to ensure completeness, internal consistency, and logical accuracy of both interview-based and recorded extracted data.

### Statistical analysis

Data were exported from the Dropbox into SPSS version 29 for cleaning and utilized Stata version 15 for analysis. Descriptive statistics, including figures, frequencies, and percentages, were computed. The study findings were presented using tables and figures. A binary and multivariate logistic regression analysis model was used to identify the relationship between dependent and independent variables. The Odds ratio was calculated, with a P-value of less than 0.05 deemed statistically significant. For further details, please refer to the published project [17]

### Ethical Approval and Informed Consent

An ethical clearance letter was obtained from the postgraduate Institutional Review Board (IRB), College of Health Sciences, Wallaga University (reference no. WU/RD/675/2023). All study steps followed the ethical rules outlined in the Declaration of Helsinki and national research ethics guidelines. For adult participants, written informed consent was obtained, and for underage participants, written assent was obtained in conjunction with informed consent from a parent or legal guardian. Permission was also received from the zonal health authorities and respective health facility managers. Confidentiality was upheld through anonymization of data, secure storage of electronic files in password-protected systems, and use of private interview settings to ensure participant safety and privacy [17].

## Result

### Sociodemographic characteristics of the study participants

The majority of the respondents are aged 19–35 years (91.7%), with Oromo being the dominant ethnic group (83.4%) and Protestantism as the most common religion (66.3%). Most participants are married (97.2%). Regarding education, 29% have attained college-level or higher, while 34.4% have completed primary education. Among partners, 43.1% have a college education or above. The primary occupation of mothers is housewives (44.7%), and most respondents reside in urban areas (82.5%). Wealth distribution is relatively balanced, with 34% categorized as rich, 33.1% as medium, and 32.5% as poor (Table 1).

**Table 1:** Socio-demographic characteristics of delivery complication assessment among pregnant women who gave birth at selected West Shoa public hospitals, Oromia, Oct 2024 (n = 573).

Variable	Categories	Frequency	Percentage
*Age*	15-18	30	5.2
	19-25	300	52.4
	26-35	225	39.3
	36-45	18	3.1
*Ethnicity (573)*	Oromo	478	83.42
	Amhara	78	13.61
	Tigray	6	1.04
	Gurage	10	1.74
	Others *	1	0.17
*Religion (573)*	Orthodox	163	28.44
	Protestant	380	66.31
	Muslim	20	3.49
	Catholic	4	0.69
	Waqefata	6	1.04
*Marital status (573)*	Never married	14	2.44
	Married	557	97.20
	Divorced/separated	2	0.34
*Educational status*	Unable to read and write	43	7.50
	Able to read and write	37	6.45
	Primary education [1–8]	197	34.38
	Secondary education	130	22.68
	College and above	166	28.97
*Educational status of partner*	Unable to read and write	26	4.53
	Able to write and read	21	3.66
	Primary education [1–8]	153	26.70
	Secondary education	126	21.98
	Tertiary education	247	43.10
*occupation*	Housewife	256	44.67
	Gov’t/private employer	166	28.97
	Merchant	100	17.45
	Student	18	3.14
	Daily labourer	25	4.40
	Others***	8	1.40
*Residence*	Urban	473	82.50
	Rural	100	17.50
*Wealth status (PCA)*	Poor	186	32.46
	Medium	192	33.50
	Rich	195	34.04

*sidama, ** Farmer.

### Maternal characteristics

The study underscores a predominantly favourable maternal and reproductive health profile among the participants. A substantial majority (85.86%) had experienced low gravidity (0–2 pregnancies), and over half (51.1%) reported short interpregnancy intervals of less than 24 months. Adverse obstetric outcomes were reported, with 4% reporting a history of low-birth-weight deliveries, 11.8% a history of abortion, and 4.7% a history of stillbirth. Remarkably, 89% of the pregnancies were intended (Table 2).

**Table 2:** Obstetrics history related characteristics of delivery complication assessment among pregnant women who gave birth at selected West Shoa public hospitals, Oromia, Oct 2024 (n = 573).

Variable	Category	Frequency	Percentage
*Gravidity*	0-2	492	85.86
	3-5	71	12.39
	>5	10	1.75
*Pregnancy interval*	Less than 24 months	293	51.1
	≥ 24 months	280	48.9
*History of having LBW*	Yes	32	4
	No	771	96
*History of abortion*	Yes	95	11.8
	No	709	88.2
*History of stillbirth*	Yes	38	4.7
	No	766	95.3
*Types of pregnancy*	Intended	510	89
	Unintended	63	11
	Mistime	29	5

### Dairy diversity consumption

**Fig 2:**
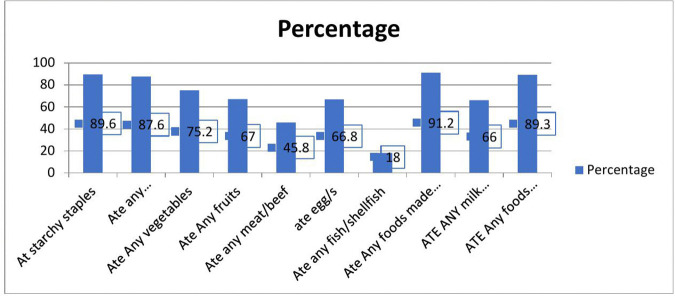
Dietary diversity consumption related to delivery complication assessment among pregnant women who gave birth at selected West Shoa public hospitals, Oromia, Oct 2024.

The mean women’s dietary diversity score of study participants was 5.03 (± 1.4SD). Among the participants, 448 (55.7%) and 356 (44.3%) had adequate and inadequate dietary diversity scores, respectively. The data show that the highest proportions of women consumed foods made with oil, fat, or sugar (91.2%), starchy staples (89.6%), other processed foods (89.3%), and potatoes or root vegetables (87.6%). Moderate consumption was observed for vegetables (75.2%), fruits (67%), eggs (66.8%), and milk products (66%). In contrast, lower consumption was recorded for meat or beef (45.8%) and especially for fish or shellfish, which had the lowest intake at 18% (Fig 2).

### Magnitude of complications

**Fig 3:**
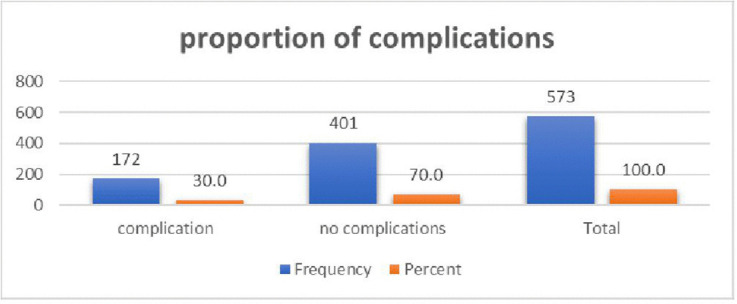
Proportion of complications among pregnant women who gave birth at selected West Shoa public hospitals, Oromia, Oct 2024.

A significant portion, 172(30%, 95%CI:26.4–33.9), experienced at least one type of complication during delivery (Fig 3).

**Fig 4:**
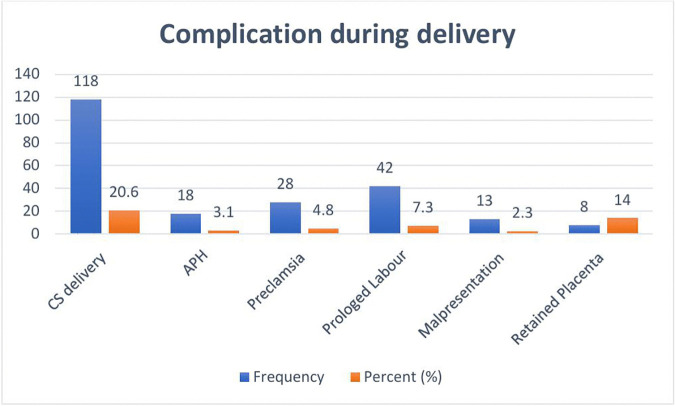
Proportions of complications observed during delivery among pregnant women who have followed ANC at West Shoa Hospitals, Oromia, Ethiopia, 2024.

Among the women surveyed, the majority (78.4%) delivered vaginally, while 20.6% underwent caesarean section (CS), and 1% had instrumental deliveries. The overall rate of caesarean section was 20.6%. Antepartum haemorrhage (APH) was reported in 3.1% of participants, and 7.3% experienced prolonged Labour. Preeclampsia affected 4.9% of the women, while retained placenta occurred in 1.4% of cases. Postpartum haemorrhage (PPH) was reported in 5.5% of the women. These findings provide an overview of maternal complications and delivery modes among pregnant women in the study population (Fig 4).

### Factors associated with complications during delivery

After controlling for potential confounders, only a small number of factors showed statistically significant independent correlations with delivery complications in multivariate logistic regression analysis. Depression during pregnancy was significantly associated with an increased risk of delivery complications. The risk of delivery complications was 38.5% greater for women who had reported depressive symptoms than for those who didn’t have it (AOR = 1.385, 95% CI: 1.059–1.81; P = 0.017)

Conversely, HIV-positive status was significantly associated with reduced odds of delivery complications. HIV-positive women were 63% less likely to experience complications compared to their HIV-negative counterparts (AOR = 0.374; 95% CI: 0.191–0.72; p = 0.004).

Anaemia was also significantly associated with delivery complications. Women without anaemia had a 6% reduced likelihood of experiencing complications compared to anaemic women (AOR = 0.94; 95% CI: 0.93–0.97; p = 0.001), suggesting that anaemia may contribute to adverse delivery outcomes.

Other factors such as low mid-upper arm circumference (MUAC < 23 cm) (AOR = 1.211; p = 0.339), alcohol use during the current pregnancy (AOR = 0.641; p = 0.39), short pregnancy interval (<24 months) (AOR = 1.184; p = 0.577), history of low birth weight (LBW) delivery (AOR = 1.526; p = 0.289), history of abortion (AOR = 1.018; p = 0.952), and history of stillbirth (AOR = 0.739; p = 0.414) were not significantly associated with delivery complications, as their confidence intervals included 1 and p-values were greater than 0.05 (Table 3).

**Table 3:** Multivariate logistic regression analysis of factors associated with delivery complications (n = 573).

	Category	COR (95%CI)	AOR (95%CI)		P-Value
Depression during pregnancy	Yes	1.52	1.385	(1.059,1.81)	0.017*
	No	1	1		
MUAC (Anthropometric measurement)	<23 cm	1.01	1.211	(0.818,1.79)	0.339
	>23	1	1		
HIV Status	Positive	0.73	0.374	(0.191,0.72)	0.004*
	Negative	1	1		
Alcohol use during pregnancy	Yes	0.47	0.641	(0.232,1.77)	0.39
	No	1	1		
Pregnancy interval	< 24 months	0.78	1.184	(0.654,2.14)	0.577
	>24 months	1	1		
History of LBW delivery	Yes	1.125	1.526	0.699,3.33)	0.289
	No	1	1	(0.96,1.35)	0.142
History of abortion	Yes	1.13	1.018	(0.563,1.84)	0.952
	No	1	1		
History of stillbirth	Yes	1.12	0.739	(0.357,1.52)	0.414
Anaemia	Yes	1	1		
	No	0.94	0.94	(0.93,0.97)	0.001*
Malpresentation	Yes	8.18	6.91	(1.79,26.68)	0.005*
	No	1	1		

## Discussion

The study assessed the proportion of delivery complications and their associated factors among pregnant women who delivered at West Shoa hospitals in the Oromia region, Ethiopia. In the present study, approximately 30% of pregnant women experienced delivery-related complications. Delivery complications are prevalent in the West Shoa Zone, with depression during pregnancy, anaemia, and malpresentation identified as significant risk factors.

This finding is consistent with a study conducted in Northern Ethiopia, where around 28% of women developed complications during delivery. The similarity may be attributed to comparable maternal health profiles, healthcare access, and obstetric care practices in both regions. However, the prevalence observed in this study is higher than that reported in a survey conducted in Jimma Zone, Dedo Woreda [23]. One plausible explanation for this discrepancy is the difference in study design. The study conducted in Jimma was a community-based, retrospective survey, which is more susceptible to recall bias and underreporting, as women may not accurately remember or disclose past complications. In contrast, the current study was facility-based, where data were collected in real-time or from medical records, likely providing a more accurate and complete picture of delivery-related complications.

Moreover, facility-based studies tend to capture cases with more severe or documented complications, as these women are more likely to seek or be referred for institutional delivery. Community-based studies, on the other hand, may miss women who delivered at home or in informal settings, where complications might go undocumented. Differences in maternal health-seeking behaviour, access to emergency obstetric services, and the quality of antenatal care could also contribute to the variation in complication rates. These findings underscore the importance of considering study methodology and context when interpreting maternal health data and highlight the need to strengthen both community outreach and facility-based maternal health services to improve outcomes.

Pregnant women who reported depressive symptoms had 38.5% higher odds of experiencing complications compared to their non-depressed counterparts (AOR = 1.385; 95% CI: 1.059–1.81; p = 0.017). This finding aligns with existing literature suggesting that maternal mental health plays a crucial role in pregnancy outcomes. A study conducted in Gondar, Ethiopia, found that women with antenatal depression had a 3.22 times higher risk of stillbirth compared to those without depression [24]. Similarly, research in rural Ethiopia indicated that antenatal depressive symptoms were linked to increased non-scheduled antenatal care visits and pregnancy-related emergency visits, suggesting a potential association with perinatal complications [25]. It can negatively impact maternal behaviour, such as reduced antenatal care attendance, poor nutrition, substance use, and limited engagement in self-care, all of which contribute to adverse maternal and neonatal outcomes [26,27]. Furthermore, physiological stress responses associated with depression, including increased levels of cortisol and inflammatory markers, may disrupt normal pregnancy processes, potentially leading to complications such as preterm labour, hypertensive disorders, or prolonged labour [28,29]. The observed association underscores the importance of integrating mental health screening and support into routine antenatal care services. Early identification and management of maternal depression could be an effective strategy to reduce the risk of delivery complications and improve overall maternal health outcomes.

Interestingly, the study found that HIV-positive status was significantly associated with reduced odds of delivery complications. HIV-positive women were 63% less likely to experience complications compared to their HIV-negative counterparts. While some studies have reported higher rates of adverse pregnancy outcomes among HIV-positive women, others have found no significant difference or even lower rates of certain complications. This finding may seem counterintuitive, but it aligns with some emerging evidence suggesting that HIV-positive women enrolled in prevention and care programs often receive more frequent and structured antenatal care, including regular monitoring, nutritional support, and access to skilled birth attendants [30]. For instance, a study in Ethiopia reported that perinatal mortality was lower among HIV-positive women compared to HIV-negative women [31].These women are often prioritized in healthcare systems due to their status, ensuring early identification and management of potential complications. Moreover, HIV-positive mothers are more likely to deliver in healthcare facilities under the supervision of trained personnel, which reduces the risk of undetected and unmanaged complications [32]. Another possible explanation is that these women may adhere more strictly to medical advice out of concern for both their health and the risk of vertical transmission, leading to better maternal outcomes.

The present study found that anaemia was significantly linked to a higher risk of delivery complications. Women without anaemia were less likely to experience complications compared to those who were anaemic, suggesting that maternal anaemia significantly contributes to adverse delivery outcomes. This aligns with studies conducted in Ethiopia and other African countries, where anaemia during pregnancy has been consistently connected to increased risks of complications such as postpartum haemorrhage, preterm birth, and low birth weight. For example, a study in the Tigray region of Ethiopia reported that anaemic pregnant women were much more likely to encounter obstetric complications than non-anaemic women. Similarly, research from Southern Ethiopia indicated that anaemia was a key predictor of poor maternal outcomes during delivery. [33].

In the broader African context, anaemia remains a major public health issue due to high rates of malnutrition, parasitic infections, and limited access to iron supplementation during pregnancy. Studies from countries such as Nigeria and Ghana have also shown that maternal anaemia is linked to increased maternal morbidity and poor delivery outcomes [34,35]. The biological mechanism may be attributed to reduced oxygen-carrying capacity in the blood, which compromises both maternal and foetal well-being and increases susceptibility to complications such as uterine inertia and prolonged labour. These findings reinforce the need for strengthening antenatal care services, particularly in screening and treating anaemia through nutritional counselling, iron supplementation, and managing underlying causes such as parasitic infections and malnutrition. Early identification and correction of anaemia could be critical in reducing the burden of maternal complications across Ethiopia and the region at large.

Malpresentation emerged as a strong and statistically significant predictor of delivery-related complications in the present study, increasing the odds of adverse outcomes. This finding is consistent with a robust body of literature that identifies foetal malpresentation particularly breech, transverse, and compound presentations, as a major obstetric risk factor, especially in low-resource settings [36,37]. Malpresentation interferes with the physiological process of Labour and delivery, increasing the likelihood of prolonged or obstructed Labour, foetal distress, uterine rupture, and postpartum haemorrhage [6,38].

In resource-constrained health systems such as those in many parts of Ethiopia and sub-Saharan Africa, the challenges of managing malpresentation are often compounded by limited access to timely caesarean delivery, skilled birth attendants, and advanced foetal monitoring. For instance, a study conducted at Debre Tabor Hospital in Northwest Ethiopia found that breech presentation was associated with significantly higher rates of neonatal asphyxia and maternal morbidity [39]. Similarly, a Nigerian study observed that malpresentation was responsible for a considerable proportion of perinatal deaths and emergency caesarean sections, underscoring its impact on both maternal and neonatal outcomes [40].

The underlying causes of malpresentation may include uterine anomalies, multiparity, polyhydramnios, foetal abnormalities, and placenta previa, which themselves are associated with a heightened risk of complications [41]. In addition, late or inadequate antenatal care reduces the likelihood of early detection and proper management of malpresentation. A study in southern Ethiopia reported that women who did not receive ultrasound screening during the third trimester were more likely to experience undiagnosed malpresentation at term [42]. Given these findings, the high odds ratio observed in this study reinforces the need for routine screening and early detection of foetal presentation during antenatal care

Other variables, including mid-upper arm circumference (MUAC) less than 23 cm, alcohol use during pregnancy, short pregnancy intervals, and histories of low-birth-weight delivery, history of previous abortion, or stillbirth, did not show statistically significant associations in the adjusted model. However, these factors may still hold clinical relevance and warrant further investigation in larger, more diverse populations.

This study offers valuable insights into the factors associated with delivery complications among pregnant women in the West Shoa Zone, utilizing primary data from multiple hospitals and employing both bivariate and multivariate analyses to control for confounding variables. A major strength of the study is its relatively large sample size and the inclusion of various maternal, clinical, and behavioral factors. However, the study also has limitations. Its cross-sectional design prevents establishing causality, and reliance on self-reported data introduces the potential for recall and social desirability bias, particularly regarding sensitive issues such as alcohol use and depressive symptoms.

## Conclusion

This study assessed the magnitude of delivery complications and associated factors among pregnant women delivered at selected public hospitals in the West Shoa Zone, Oromia, Ethiopia. The magnitude of delivery complications is high in the current study, and the findings of the study revealed that having antenatal depressive symptoms, foetal malpresentation, and anaemia, were significantly associated with delivery complications. HIV-positive status was significantly associated with reduced odds of delivery complications. Pregnant women experiencing depressive symptoms were significantly higher risk of developing delivery complications when compared to non-depressed pregnant women. Women with low haemoglobin had lower odds of experiencing delivery complications compared with non-anaemic women. Women with foetal malpresentation had significantly increased odds of experiencing delivery complications compared with cephalic presentation. HIV positive women exhibited a significantly lower risk of delivery complications compared to HIV negative women

While other factors like having low mid-upper arm circumferences (MUA), short pregnancy intervals, alcohol consumption, and previous history of adverse birth outcome did not show a statistically significant association in the multivariate analysis model, their clinical relevance should not be overlooked.

### Recommendations

The study provided recommendations to reduce the burden of delivery-related complications in the study area and beyond. The necessity of incorporating mental health screening and integrating it into ANC services is fundamental in reducing prenatal depression and birth complications. A trained health care provider should identify and manage it to mitigate its impact on maternal outcomes.

Furthermore, strengthening nutritional counselling to prevent anaemia, routine haemoglobin screening, and promoting adherence to Iron and Folic Acid supplementation (IFA 90+). Although an HIV-positive status was associated with reduced complications—likely due to structured follow-up—this suggests a need to extend similar comprehensive care and follow-up mechanisms to all pregnant women, regardless of HIV status.

The importance of a third-trimester ultrasound for all pregnant women to diagnose timely and to arrange referral systems to skilled obstetric management for malpresentation.

Lastly, future research using longitudinal designs is recommended to explore the temporal and causal relationships between these factors and delivery outcomes.

## Supporting information

S1 File Raw data for delivery complications. (XLSX)
